# Indirect effect of temperature on fish population abundances through phenological changes

**DOI:** 10.1371/journal.pone.0175735

**Published:** 2017-04-18

**Authors:** Lucie Kuczynski, Mathieu Chevalier, Pascal Laffaille, Marion Legrand, Gaël Grenouillet

**Affiliations:** 1 EDB (Laboratoire Évolution & Diversité Biologique), CNRS, Université Toulouse 3 Paul Sabatier, Toulouse, France; 2 EcoLab, INP, UPS, ENSAT, Castanet Tolosan, France; 3 Logrami (Loire Grands Migrateurs), Orléans, France; 4 Institut Universitaire de France, Paris, France; Helmholtz-Zentrum fur Ozeanforschung Kiel, GERMANY

## Abstract

In response to climate change, earlier phenological events have been reported for a large range of taxa such that phenological shifts are considered as one of the fingerprints of the effect of climate change on organisms. Evidence further suggests that changes in the timing of phenological events might decouple biotic interactions due to differential phenological adjustment among interacting species, ultimately leading to population declines. Nonetheless, few studies have investigated how climate-driven changes in the timing of phenological events influence population abundances. In this study, we investigated how two environmental variables known to influence the migration timing of freshwater fish (i.e. water discharge and temperature) directly or indirectly influenced abundances of 21 fish species using daily time series gathered at four sites located in France over a period spanning from 9 to 21 years. We found no evidence for long-term trends in migration timing or fish abundances over time. Using piecewise structural equation models, we demonstrate that inter-annual variations in abundances were driven by inter-annual variations in temperature through variations in migration timing. Overall, our results suggest that climate change may concomitantly influence different biological aspects (e.g. phenology, abundance) of fish species. We argue that considering different responses to climate change is paramount if we are to improve our understanding of how organisms and populations are influenced by climate change in order to set-up efficient conservation strategies.

## Introduction

Over the past few years, many studies have reported that climate change has modified a large set of environmental parameters (e.g. temperature, rainfall, hydrological and fire regimes) with varying impacts on organisms, populations, communities and ecosystems [1–3]. Facing climate change, species exhibit a wide range of responses, which are associated to different processes. For instance, climate change can influence organisms through their physiological activity [4]. Likewise, various studies have reported a direct influence of temperature on several demographic parameters (e.g. mortality rate, reproductive success) with consequences on population abundances and population dynamics [2,5]. Another response to climate change is species distribution changes, with shifts poleward in latitude, upward in elevation or deeper in depth for marine organisms [3,6,7]. These geographical range changes are caused by local population extinctions at the trailing edge of the distribution, and population colonization at the leading edge with variable changes in local abundances within the core of the distribution [8]. Such extinction and colonization events represent a direct impact of climate change on local abundances where the performance of populations is enhanced or reduced because of changes in local environmental conditions at the boundaries of the species range [4]. However, indirect influences of climate change on population abundances have recently been addressed [9,10].

Phenology (i.e. the timing of seasonal activities such as migration, flowering or breeding) is a trait that is highly sensitive to climate warming [8,11,12] because temperature is an essential trigger of phenological events such as migration [13] due to its influence on physiological and behavioral processes [14]. Furthermore, phenology is a trait that is particularly important because it determines the reproductive success, survivorship and fitness of many species [15,16]. Over the past few years, several studies have shown that failure to adjust the timing of life-cycle events to climate may jeopardize populations by causing ecological mismatches to the life cycle of other species and abiotic factors [17,18]. For instance, it has been suggested that population declines of some migratory birds breeding in Europe result from their inability to adjust migration phenology to keep track of advancement of spring events at their breeding grounds [19,20]. Consequently, climate change may have indirect consequences on population abundances by causing mismatches in the timing of phenological events [19,21].

In the global warming context, ectothermic organisms are expected to respond more sharply to temperature variations than endothermic organisms because their metabolism is directly dependent on the environment [22]. Among ectothermic organisms, aquatic species are especially sensitive to climate change because their ecosystems are among the most affected [1]. As a consequence, marine fish species such as the herring (*Clupea harengus*) [23,24] and anadromous species such as the Atlantic Salmon (*Salmo salar*) [13,25] have received increasing attention. Nonetheless, ecological responses of fish species to global warming and more specifically phenological changes in the timing of migration have been poorly investigated relative to other taxonomic groups [26]. Furthermore, the studies conducted so far on fishes have mainly focused on marine species recognized for their high commercial and/or cultural values such as anadromous salmonids [13]. Surprisingly, very few studies have simultaneously investigated the influence of climate change on the phenology and abundance of freshwater fish species, even though these species play a major role in the functioning of freshwater ecosystems [27,28]. Yet understanding how freshwater fish populations respond to environmental changes and how phenological changes, such as shifts in the timing of migration, influence the abundances of these populations is essential if we are to predict the impact of future climate change on the sustainability of freshwater species and set-up efficient conservation strategies for freshwater ecosystems.

In this study, we explored the joint temporal dynamics of freshwater fish phenology and abundances using migratory time series data gathered at fish passes of four dams located in France. We specifically investigated whether temperature and water discharge had an impact on the phenology and abundances of 51 populations from 21 freshwater fish species and tested whether these environmental variables influenced population abundances (i.e. the number of individuals that migrate) directly or indirectly through an influence on the phenology (i.e. the timing of migration).

## Material and methods

### Study sites and environmental variables

**Fig 1:**
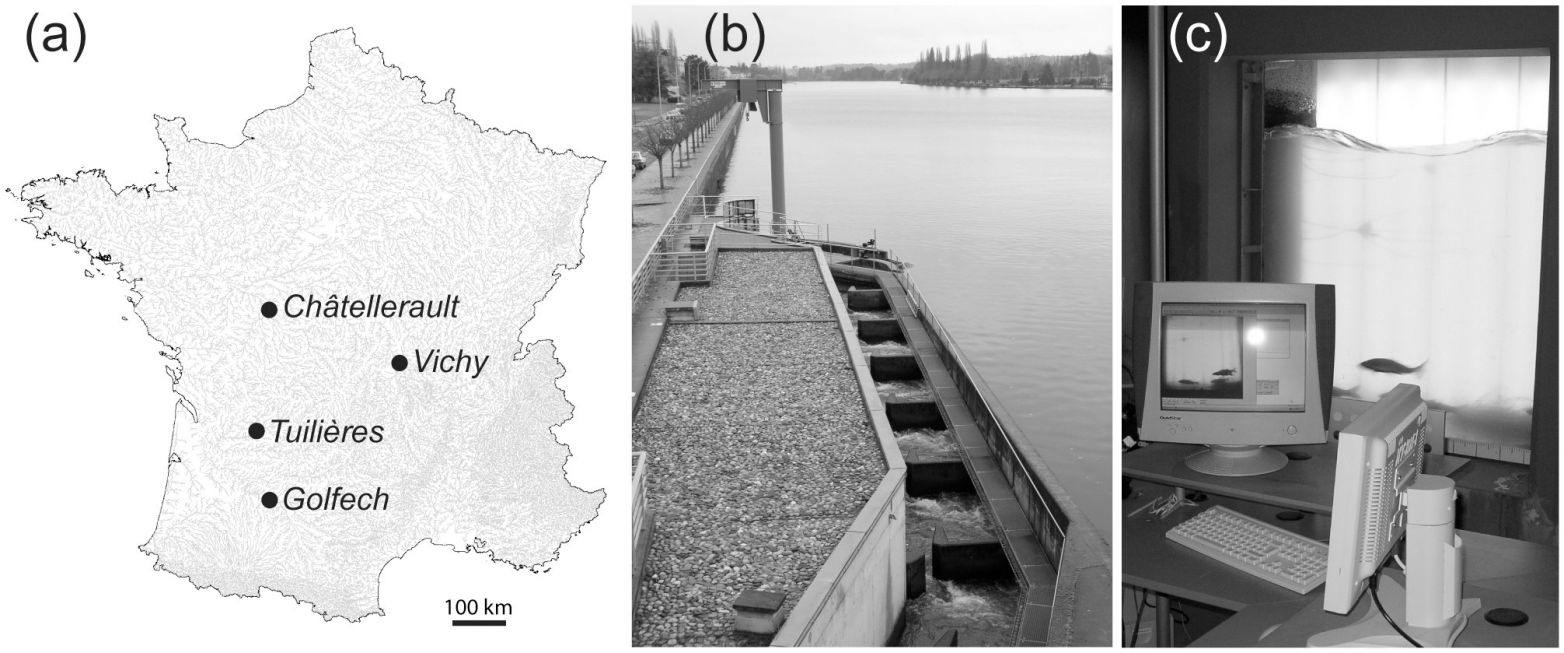
Location of studied dams. (a) Points are studied dams. (b) Fish ladder at Vichy where (c) video station is used to identify and count fishes.

We used daily time series gathered at four dams located in France (Fig 1), including environmental data (water temperature (°C) and river discharge (m^3^ *per* second)) and count data for several freshwater fish species. Dams at Châtellerault and Vichy are located in the Loire River whereas dams at Tuilières and Golfech are located in the Dordogne and the Garonne River, respectively. Time series at Châtellerault started in 2004 and ended in 2012 (9-year time series) whereas the one at Vichy started in 1997 and ended in 2012 (16-year time series). Time series at both Golfech and Tuilières started in 1993 and ended in 2013 (21-year time series). All dams have a run-of-the-river functioning, with little impact on natural river discharge. Temperature data have been recorded hourly at 0.5 to 2 meters in depth, depending on the dam and then averaged in order to obtain daily mean temperatures. Discharge data have also been recorded hourly and averaged over the day [29]. Fish counts have been gathered using continuous video analysis with the software SYSIPAP. All permissions to collect data were obtained from DREAL.

From environmental data, we computed six variables that were used to describe environmental conditions at each site and in each year: mean winter temperature and discharge (calculated from December of the previous year to February), mean spring temperature and discharge (from March to May), and mean summer temperature and discharge (from June to August). When temperature time series had periods with missing values (e.g. because of a sensor breakdown), values were interpolated with Fourier transforms. We chose this method to take into account the cyclic annual component of temperature data. When the time series of river discharges had missing values we used linear interpolations instead of Fourier transforms because the latter method is not relevant for detecting abrupt changes that are typical of hydrological data. Overall, the total length of gaps in time series represented, at a maximum, 3% of the length of the considered time series. The longest observed gap was for temperature at Vichy and lasted 2 months. On average, gaps lasted 17 days (sd = 18 days).

### Biological time series

To study population abundances through time, we focused on the total number of individuals that migrate upstream through the dams each year.

To study the temporal changes in phenology we focused on three metrics classically used in the literature: the starting, the median and the ending date of upstream migration [30]. The median date of migration was defined as the Julian day when half of the individuals have migrated whereas the starting and the ending date of migration corresponded to the days when 5% and 95% of all individuals have migrated, respectively [30]. For each species, we removed the years where migration duration was superior to 200 days because we considered that it represented a continuous movement of the population rather than a unique life cycle phase. We also removed the years where the number of individuals was less than 20 because we considered that this number was not representative of a population. Although arbitrary, these thresholds allowed us to focus on the most biologically relevant years, while keeping enough observations to study changes in fish abundances and phenology over time. Consequently, our study was based on 21 fish species and 51 populations spread over four sampling sites: seven at Châtellerault, 17 at Golfech, 18 at Tuilières and nine at Vichy. For the Atlantic salmon (*Salmo salar*), we chose not to account for the autumnal migration as few individuals migrated during this period and those that did migrate would not generally have good reproductive success [31].

### Statistical analyses

To test how environmental (i.e. the seasonal metrics for both river discharge and water temperatures) and biological (i.e. annual population abundances, the starting, the median and the ending date of upstream migration) variables varied over time, we used for each response variable linear mixed-effects models (LMMs) with the year as an explanatory variable. For all models, the year was standardized and site identity was included as a random effect to account for spatial differences in temporal trends. For biological variables, we further included species identity as a random effect to account for interspecific differences in temporal trends. To describe species-specific temporal trends in biological variables, we performed a Principal Component Analysis (PCA) on random slopes estimated by LMMs for the four biological variables (i.e. starting, median and ending date of migration and abundances).

To account for colinearity between seasonal variables, we performed two PCAs on temperature and discharge variables, respectively. We kept the first axes of each analysis as synthetic variables representing temperature and discharge variations, accounting for 48% and 70% of the total variance, respectively. For both analyses, seasonal variables were strongly correlated with the kept axes (correlation ranging from 0.64 and 0.86 and from 0.84 and 0.92, for temperature and discharge, respectively). To determine whether environmental variables influenced freshwater fish abundances directly or indirectly through changes in phenological events, we used piecewise structural equation models (SEM, [32]) with site and species as random effects to account for spatial and specific variations regarding the effect of environmental variables on phenology and population abundances. Piecewise SEM was built with seven mixed-effects models: three with each of the three phenological metrics as dependent variable and the two PCA axes as independent variables, three with population abundances as dependent variables and each of the three phenological metrics as independent variables, and one with population abundances as dependent variable and the two PCA axes as independent variables. The relevance of random effects was assessed by the comparison of AIC [33] between SEM including or not including random effects.

Model coefficients within the piecewise SEM procedure were estimated with a restricted maximum likelihood approach. The completeness of the models was assessed by means of Fisher’s C [32]. As we did not include the relationships between phenological metrics, our SEM was considered incomplete (C = 556; P < 0.001). However, given that such relationships were not biologically relevant (it is meaningless to consider that the ending date of migration in a given year influences the starting date of the same year), we conserved the model in its current form. Prior to analyses, the number of individuals was log-transformed to meet normality assumptions.

All analyses were performed with R 3.1.3, with the packages *ade4* [34], *nlme* [35] and *piecewiseSEM* [32].

## Results

**Fig 2:**
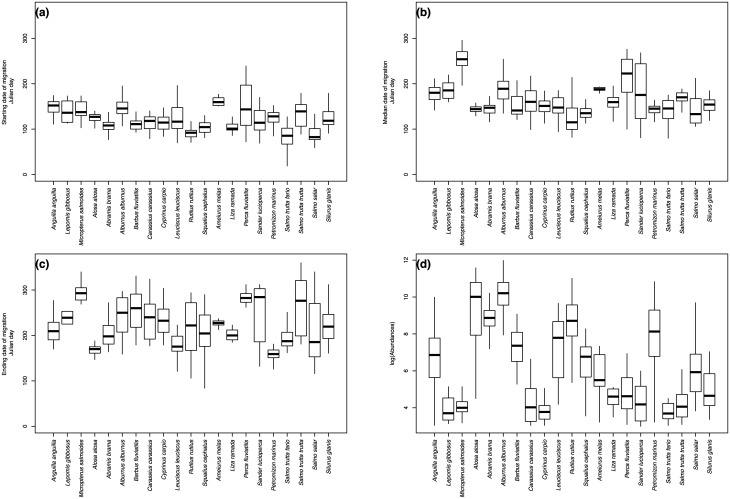
Interspecific variability in biological metrics. (a) Starting, (b) median and (c) ending dates of migration for each studied species and (d) their log-transformed abundances.

In terms of Julian days (Fig 2), the starting date of upstream migration ranged between days 4 and 248 (mean = 121, sd = 31 days) whereas the median date of migration ranged between days 70 and 307 (mean = 161, sd = 41 days). The ending date of migration ranged between days 84 and 360 (mean = 220, sd = 53 days). Finally, population abundances ranged between 3 and 12 on the log scale (mean = 7.0, sd = 2.0) that is between 20 and 159162 individuals on the natural scale (mean = 7219.3, sd = 17264.22).

### Temporal trends

Models used to investigate temporal trends in environmental time series at each site fitted the data well for both water discharge (conditional R^2^ ranging between 0.58 and 0.64) and temperature in spring and winter (conditional R^2^ equaling 0.35 and 0.62, respectively). In contrast, the conditional R^2^ was low (0.03) for summer temperature. Overall, environmental conditions remained stable over the study period (Table 1). The absence of a global long-term trend combined with the high values reported for R^2^, overall indicated that there was strong inter-site variability in temporal trends.

**Table 1:** Temporal trends in abiotic and biological variables.

		Estimate	P	R^2^
**Temperature** ***(°C*.*year***^***-1***^***)***	**Winter**	0.18	0.66	0.62
	**Spring**	0.13	1	0.35
	**Summer**	0.11	0.53	0.03
**Discharge** ***(m***^***3***^.***s***^***-1***^.***year***^***-1***^***)***	**Winter**	- 48.91	0.15	0.58
	**Spring**	11.08	0.54	0.63
	**Summer**	1.61	0.72	0.64
**Phenology** ***(days*.*year***^***-1***^***)***	**Start**	- 1.05	0.74	0.52
	**Median**	- 2.06	0.60	0.55
	**End**	- 2.19	0.61	0.49
**log(Abundances)** ***(log(individuals)*.*year***^***-1***^***)***		- 0.043	0.77	0.74

Estimates of temporal trends for each environmental and biological variable (fixed effect coefficients from linear mixed-effects models including site as random effect) with their associated p-values (P) and conditional R^2^.

**Fig 3:**
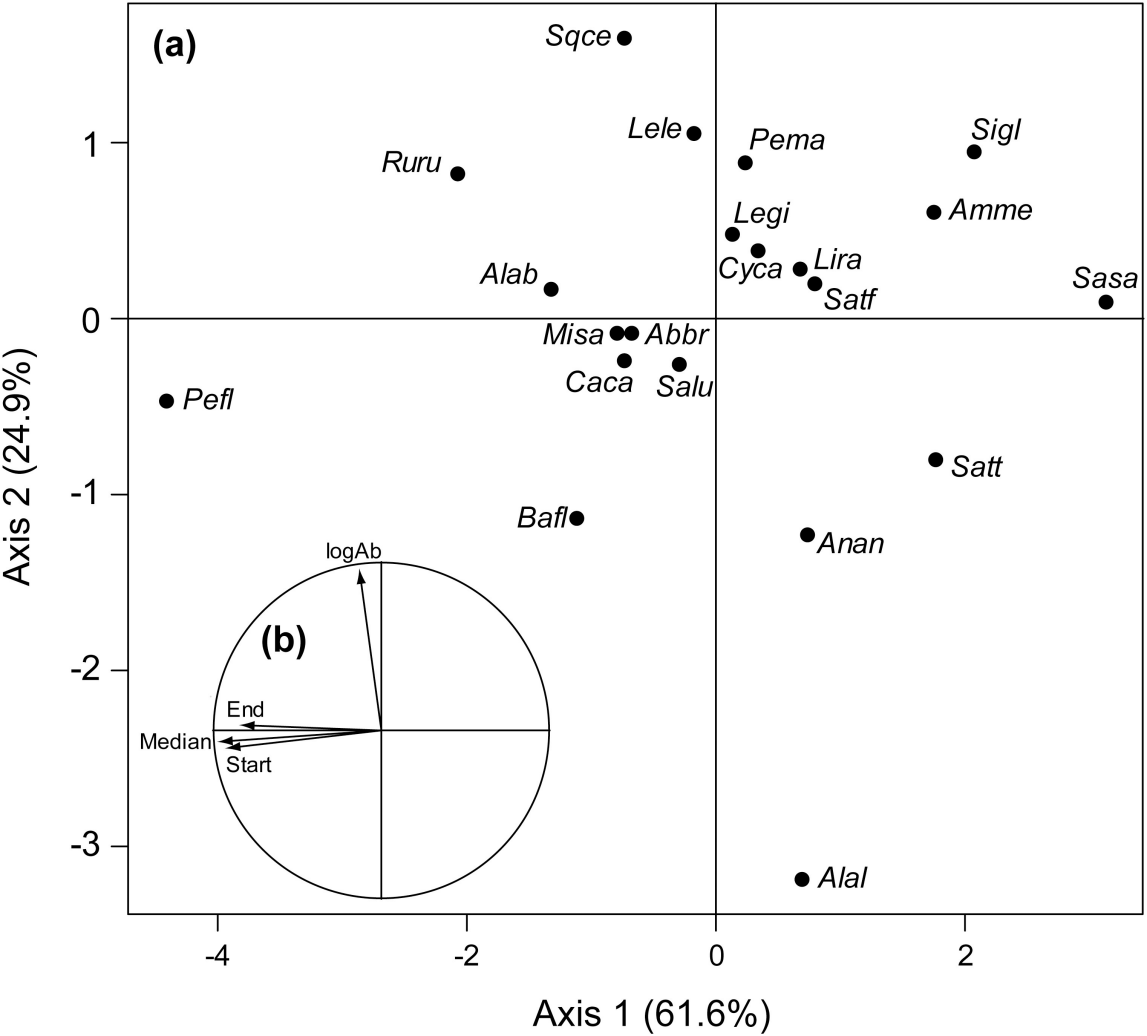
Results from the principal components analysis performed on species-specific temporal trends in the four biological variables. (a) Each point represents a species; abbreviations are given in Table 2. The first two axes explained 61.6% and 24.9% of the total variance, respectively. (b) Arrows represent the projection of the four trends in biological variables on the two dimensional space defined by the correlation circle.

The variance explained by random effects for the starting, the median and the ending date of migration ranged between 49% and 54% while the variance explained by random effects regarding log-transformed abundances reached 74%. Overall, we found no significant linear trends in each of the four biological variables (i.e. the starting, the median and the ending date of upstream migration and the annual population abundances; Table 1) over time. This lack of a global trend was the result of a large variability in species-specific coefficients. For instance, species such as *Anguilla anguilla*, *Alosa alosa* and *Salmo salar* experienced earlier migration while others species, like *Barbus fluviatilis*, *Carassius carassius* or *Perca fluviatilis*, showed delayed migration (Table 2). Similarly, we found a large variability regarding trends in population abundances. Although species like *Anguilla anguilla*, *Alosa alosa* and *Salmo trutta trutta* decreased in abundance over the study period, warm-water or exotic species, such as *Lepomis gibbosus*, *Cyprinus carpio* and *Silurus glanis*, were found to increase in abundance (Table 2). Overall, we found that the trends reported for phenological metrics were all correlated between each other but were not correlated with trends in abundances (Fig 3). Finally, species belonging to the *Salmonidae* and *Siluridae* families tended to exhibit similar responses in term of phenological shifts (i.e. earlier migration).

**Table 2:** Species-specific temporal trends in biological variables.

Family	Species Abbreviations (number of populations)	Starting date	Median date	Ending date	log(Abundances) *log(indivuduals) per year*
		*Days per year*			
**Anguillidae**	*Anguilla Anguilla* Anan (n = 3)	- 8.61	- 6.71	- 1.21	- 0.63
**Centrarchidae**	*Lepomis gibbosus* Legi (n = 1)	- 0.83	- 8.43	0.28	0.15
	*Micropterus salmoides* Misa (n = 3)	11.48	1.28	- 4.40	- 0.01
**Clupeidea**	*Alosa alosa* Alal (n = 2)	- 4.69	- 4.25	- 4.26	- 1.46
**Cyprinidae**	*Abramis brama* Abbr (n = 2)	2.25	- 0.18	6.63	- 0.07
	*Alburnus alburnus* Alab (n = 2)	6.91	7.66	6.25	0.08
	*Barbus fluviatilis* Bafl (n = 2)	1.42	6.06	12.89	- 0.53
	*Carassius carassius* Caca (n = 3)	4.00	0.31	5.36	- 0.13
	*Cyprinus carpio* Cyca (n = 4)	- 3.84	- 7.58	- 1.02	0.10
	*Leuciscus leuciscus* Lele (n = 1)	- 2.41	- 1.00	0.22	0.41
	*Rutilus rutilus* Ruru (n = 2)	5.38	19.73	11.04	0.38
	*Squalius cephalus* Sqce (n = 2)	- 2.03	- 0.39	10.57	0.64
**Ictaluridae**	*Ameiurus melas* Amme (n = 2)	- 12.07	- 16.78	- 12.40	0.15
**Mugilidae**	*Liza ramada* Lira (n = 1)	- 4.21	- 7.79	- 7.49	0.06
**Percidae**	*Perca fluviatilis* Pefl (n = 3)	30.52	40.84	10.37	- 0.02
	*Sander lucioperca* Salu (n = 3)	0.72	- 0.26	0.45	- 0.15
**Petromyzontidae**	*Petromyzon marinus* Pema (n = 4)	- 4.21	- 2.45	- 4.40	0.33
**Salmonidae**	*Salmo trutta fario* Satf (n = 2)	- 13.83	- 6.45	1.30	- 0.03
	*Salmo trutta trutta* Satt (n = 2)	- 1.49	- 15.84	-25.00	- 0.40
	*Salmo salar* Sasa (n = 3)	- 14.58	- 21.30	- 33.76	- 0.07
**Siluridae**	*Silurus glanis* Sigl (n = 4)	- 11.93	- 19.66	- 17.35	0.30

Studied species, the corresponding abbreviation of the Latin name, the corresponding number of populations and estimates of temporal trends for each biological variable (i.e. random slope coefficients from the linear mixed-effects models).

### Environmental and phenological determinants of population abundances

**Fig 4:**
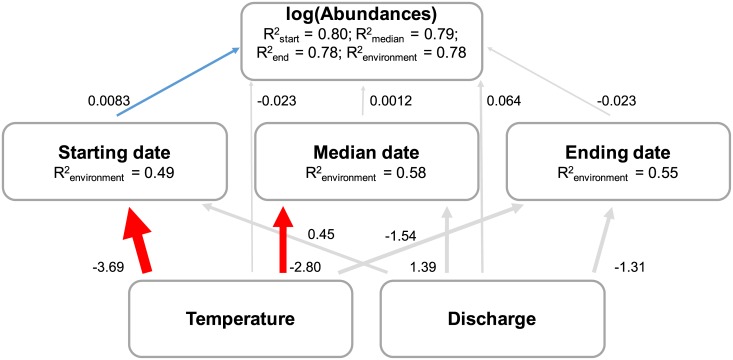
Results of the piecewise structural equation model. Blue and red lines represent significant positive and negative relationships, respectively, whereas grey lines represent non-significant relationships. Path coefficient estimates are shown alongside arrows for all tested relationships. R^2^ are provided for each of the seven models. Temperature and Discharge are synthetic variables (i.e. first axis) extracted from the two PCAs.

Based on AIC, we found that SEM including sites and species as random effects had better support relative to SEM not including random effects (ΔAIC = 168), thus suggesting that the relationships modeled within the SEM were likely to vary both spatially and among species (Tales A-G in S1 File). Water temperature and discharge conjointly explained a great amount of variance regarding the starting (49%), the median (58%), and the ending date of migration (55%). Furthermore, between 78 and 80% of the variance in population abundances were explained by environmental and phenological variables. Overall, we found a negative relationship between water temperature and phenological metrics, especially regarding the starting (estimate = -3.69; P < 0.001) and the median date of migration (estimate = -2.80; P = 0.034), indicating that higher temperatures induced earlier migration (Fig 4). We also found a significant positive effect of the starting date of migration (estimate = 0.0083; P < 0.001) on population abundances (Fig 4). Thus, the earlier the migration is, the greater the population declined. Overall, these results revealed an indirect effect of water temperature on demography through its influence on the starting date of migration (-3.69 x 0.0083 = - 0.031) which corresponded to population declines with increased temperatures (Fig 4).

## Discussion

In this study, our aim was (1) to quantify temporal trends in environmental variables, phenology and population abundances, and (2) to determine whether inter-annual fluctuations in climate influenced fish population abundances directly or indirectly through an influence on the migration timing. We did not found any evidence for temporal trends in water temperature and river discharge in French rivers within the study period. Regarding the timing of migration events and population abundances, no significant long-term trends were observed. However, we observed that temporal variations in abundances and/or timing in migration were highly variable in terms of direction and/or intensity depending on the considered species. Finally, we demonstrated that inter-annual variations in abundances were driven by inter-annual variations in temperature through variations in migration timing.

Phenological plasticity in response to environmental change has been reported for several taxa, including trees [36–38], birds [39,40], mammals [41], amphibians and reptiles [42]. Our results add to this knowledge by providing evidence that stream fish species are able to track climate fluctuations by altering the phenology of migration from year to year. Moreover, we found an indirect and negative influence of temperature on abundances, through earlier migration timing as previously found [43]. This suggests that even though species are able to detect environmental changes, their response may not be sufficient to cope with the changes they experience. Such lag in the response could ultimately lead to population declines by causing ecological mismatches to the life cycle of other species and abiotic factors. For instance, several studies have reported a decoupling between the emergence date of insects and the peak of food availability leading to a reduction in insect density [44,45]. Decoupled interactions at low trophic level are especially problematic because they are likely to propagate along the food chain and to also impact species at higher trophic levels [19,45,46].

Our study also highlights the need to address both direct and indirect effects of the environment if we are to improve our understanding of the influence of climate change on population dynamics. Indeed, our modeling framework revealed an indirect influence of temperature on population abundances through its influence on the starting date of migration. Specifically, we found that although stream fish species are able to track climate variation by adapting their migration timing, populations could still decline. This result was quite unexpected because as species adapt their phenology, populations should remain within suitable environmental conditions and thus remain stable. Two non-mutually exclusive hypotheses can explain this pattern. First, phenological changes may not have been strong enough to keep pace with local changes in environmental conditions, thus leading to population declines. Second, species may have changed their phenology in accordance with environmental conditions differently to their associated prey, leading to trophic mismatches and therefore population declines.

Although it is widely accepted that species are able to respond to climate change through changes in distribution, phenology and physiology [4,11,47], many studies have reported a delay in these biological responses [48–50]. This suggests that species may not be able to track changes in environmental conditions fast enough. What are the processes responsible for these lags and what are their ecological consequences are still open questions deserving attention in the future. For instance, there is growing evidence that the response of entire communities is lagging behind climate change [48,51,52] and it would be interesting to determine how this lag is affecting ecosystem processes. Likewise, species exhibiting important climatic debt may be more vulnerable to environmental modifications, which could lead to a loss of taxonomic or functional biodiversity. Such loss can ultimately decrease ecosystem resistance and/or resilience and lead to a loss of essential ecosystem functions [53–55]. Given that freshwater ecosystems provide important services to human societies and are among the most threatened by climate changes, further studies are clearly needed to improve our knowledge regarding the mechanisms through which climate is influencing species within these ecosystems [56].

## Supporting information

S1 File Outputs of mixed effects models used within SEM. Estimated coefficients and standard errors of mixed effects models. For random effects, the values of Intercept and Slope correspond to the estimated variance (and its associated standard deviation) around the fixed effects due to species and populations.
(DOCX)

S2 File Abiotic data. Daily temperature and discharge data for the four studied sites.
(XLSX)
