# Supplementary material for: Indirect effect of temperature on fish population abundances through phenological changes
Source: PLoS One. 2017 Apr 18;12(4):e0175735. doi: 10.1371/journal.pone.0175735 (PMC5395187; doi:10.1371/journal.pone.0175735)
Supplement: S1 File — Estimated coefficients and standard errors of mixed effects models. For random effects, the values of Intercept and Slope correspond to the estimated variance (and its associated standard deviation) around the fixed effects due to species and populations. (DOCX) [file pone.0175735.s001.docx]

*Appendix to:*

Lucie Kuczynski, Mathieu Chevalier, Pascal Laffaille, Marion Legrand and Gaël Grenouillet (2016) Indirect Effect of Temperature on Fish Population Abundances Through Phenological Changes. *Submitted paper*.

*Appendix S1*

Outputs of mixed effects models used within SEM.

**Table A.** Estimated coefficients and standard errors of mixed effects model relating Abundances (response variable in log) to the starting date of migration (explanatory variable). For random effects, the values of Intercept and Slope correspond to the estimated variance (and its associated standard deviation) around the fixed effects due to species and populations.

| **Explanatory variable** | **Fixed effect** | | **Random effect: Species** | | **Random effect: Population** | |
| --- | --- | --- | --- | --- | --- | --- |
|  | **Intercept** | **Slope** | **Intercept** | **Slope** | **Intercept** | **Slope** |
| **Starting date of migration** | 5.01 (0.52) | 0.0083 (0.002) | 3,83 (1.96) | 2,58 10^-4^ (0.016) | 0,85 (0.91) | 6,81 10^-5^ (0.0083) |

**Table B.** Estimated coefficients and standard errors of mixed effects model relating Abundances (response variable in log) to the median date of migration (explanatory variable). For random effects, the values of Intercept and Slope correspond to the estimated variance (and its associated standard deviation) around the fixed effects due to species and populations.

| **Explanatory variable** | **Fixed effect** | | **Random effect: Species** | | **Random effect: Population** | |
| --- | --- | --- | --- | --- | --- | --- |
|  | **Intercept** | **Slope** | **Intercept** | **Slope** | **Intercept** | **Slope** |
| **Median date of migration** | 5.83 (0.53) | 0.0012 (0.0017) | 3,86 (1.96) | 1,41 10^-4^ (0.012) | 0,77 (0.88) | 3,52 10^-5^ (0.0059) |

**Table C.** Estimated coefficients and standard errors of mixed effects model relating Abundances (response variable in log) to the ending date of migration (explanatory variable). For random effects, the values of Intercept and Slope correspond to the estimated variance (and its associated standard deviation) around the fixed effects due to species and populations.

| **Explanatory variable** | **Fixed effect** | | **Random effect: Species** | | **Random effect: Population** | |
| --- | --- | --- | --- | --- | --- | --- |
|  | **Intercept** | **Slope** | **Intercept** | **Slope** | **Intercept** | **Slope** |
| **Ending date of migration** | 6.51 (0.53) | -0.023 (0.0013) | 3,67 (1.92) | 7,30 10^-5^ (0.0085) | 0,75 (0.87) | 1,81 10^-5^ (0.0043) |

**Table D.** Estimated coefficients and standard errors of mixed effects model relating Abundances (response variable in log) to the temperature and discharge (explanatory variables). For random effects, the values of Intercept and Slope correspond to the estimated variance (and its associated standard deviation) around the fixed effects due to species and populations.

| **Explanatory variable** | **Fixed effect** | | **Random effect: Species** | | **Random effect: Population** | |
| --- | --- | --- | --- | --- | --- | --- |
|  | **Intercept** | **Slope** | **Intercept** | **Slope** | **Intercept** | **Slope** |
| **Temperature** | 6.02 (0.46) | -0.023 (0.049) | 3,90 (1.98) | 4,26 10^-9^ (6.53 10^-5^) | 0,79 (0.89) | 1,27 10^-1^ (1.19) |
| **Discharge** |  | 0.064 (0.061) |  | 1.34 10^-9^ (0.00012) |  | 1,21 (0.36) |

**Table E.** Estimated coefficients and standard errors of mixed effects model relating the starting date of migration to the temperature and discharge (explanatory variables). For random effects, the values of Intercept and Slope correspond to the estimated variance (and its associated standard deviation) around the fixed effects due to species and populations.

| **Explanatory variable** | **Fixed effect** | | **Random effect: Species** | | **Random effect: Population** | |
| --- | --- | --- | --- | --- | --- | --- |
|  | **Intercept** | **Slope** | **Intercept** | **Slope** | **Slope** | **Intercept** |
| **Temperature** | 122.17 (4.68) | -3.69 (1.05) | 366 (19.13) | 4,83 10^-7^ (0.0007) | 136 (11.65) | 41,7 (6.46) |
| **Discharge** |  | 0.45 (1.23) |  | 2,21 10^-5^ (0.0047) |  | 189 (13.76) |

**Table F.** Estimated coefficients and standard errors of mixed effects model relating the median date of migration to the temperature and discharge (explanatory variables). For random effects, the values of Intercept and Slope correspond to the estimated variance (and its associated standard deviation) around the fixed effects due to species and populations.

| **Explanatory variable** | **Fixed effect** | | **Random effect: Species** | | **Random effect: Population** | |
| --- | --- | --- | --- | --- | --- | --- |
|  | **Intercept** | **Slope** | **Intercept** | **Slope** | **Slope** | **Intercept** |
| **Temperature** | 165.87 (6.50) | -2.80 (1.32) | 655 (25.58) | 5,80 10^-9^ (7.61 10^-5^) | 408 (20.19) | 8,38 10^-6^  (0.0029) |
| **Discharge** |  | 1.39 (1.64) |  | 78,7 (8.87) |  | 482 (21.95) |

**Table G.** Estimated coefficients and standard errors of mixed effects model relating the ending date of migration to the temperature and discharge (explanatory variables). For random effects, the values of Intercept and Slope correspond to the estimated variance (and its associated standard deviation) around the fixed effects due to species and populations.

| **Explanatory variable** | **Fixed effect** | | **Random effect: Species** | | **Random effect: Population** | |
| --- | --- | --- | --- | --- | --- | --- |
|  | **Intercept** | **Slope** | **Intercept** | **Slope** | **Slope** | **Intercept** |
| **Temperature** | 224.21 (7.94) | -1.54 (1.58) | 953 (30.86) | 5,13 10^-5^ (0.0072) | 640 (25.30) | 82,3 (9.08) |
| **Discharge** |  | -1.31 (1.88) |  | 69,5 (8.34) |  | 353 (18.8) |
